# Active Low Intrusion Hybrid Monitor for Wireless Sensor Networks

**DOI:** 10.3390/s150923927

**Published:** 2015-09-18

**Authors:** Marlon Navia, Jose C. Campelo, Alberto Bonastre, Rafael Ors, Juan V. Capella, Juan J. Serrano

**Affiliations:** 1 Computer Science School, Escuela Superior Politécnica Agropecuaria de Manabí (ESPAM MFL), 10 de Agosto and Granda Centeno Street, No. 82, 130607 Calceta, Ecuador; E-Mail: mnaviam@espam.edu.ec; 2 IUI ITACA, Sensor Networks Lab, Universitat Politècnica de València, Camino de Vera, s/n, 46022 Valencia, Spain; E-Mails: jcampelo@itaca.upv.es (J.C.C.); bonastre@itaca.upv.es (A.B.); rors@itaca.upv.es (R.O.); jserrano@itaca.upv.es (J.J.S.)

**Keywords:** monitoring, hybrid monitor, intrusion, wireless sensor networks

## Abstract

Several systems have been proposed to monitor wireless sensor networks (WSN). These systems may be active (causing a high degree of intrusion) or passive (low observability inside the nodes). This paper presents the implementation of an active hybrid (hardware and software) monitor with low intrusion. It is based on the addition to the sensor node of a monitor node (hardware part) which, through a standard interface, is able to receive the monitoring information sent by a piece of software executed in the sensor node. The intrusion on time, code, and energy caused in the sensor nodes by the monitor is evaluated as a function of data size and the interface used. Then different interfaces, commonly available in sensor nodes, are evaluated: serial transmission (USART), serial peripheral interface (SPI), and parallel. The proposed hybrid monitor provides highly detailed information, barely disturbed by the measurement tool (interference), about the behavior of the WSN that may be used to evaluate many properties such as performance, dependability, security, *etc*. Monitor nodes are self-powered and may be removed after the monitoring campaign to be reused in other campaigns and/or WSNs. No other hardware-independent monitoring platforms with such low interference have been found in the literature.

## 1. Introduction

Wireless sensor networks (WSN) have been the subject of significant research and development in recent years. However they are yet to be deployed on a mass scale because sensor networks may experience problems or errors in their operation. Many causes for such issues have been identified, such as interference in the transmission medium, security attacks (especially in WSN [1]), adverse environmental conditions, and malfunctioning nodes. The node faults, their sources, and detection approaches are diverse, as detailed in [2]. Although debugging and operations testing is usually carried out during the development and implementation of this type of network, when sensors are deployed the conditions may be very different and unanticipated events often occur.

The availability of suitable sensor network diagnostic tools is a key issue in progressing to real-world deployment of WSN. Nowadays, there are no standard tools or standard architectures in this field. Most of the proposals for monitoring and debugging do not consider enough aspects of sensor networks to be fully useful or are built for very specific network architectures. According to [3], there are many challenges in several aspects of sensor networks—architectural, functional, and dynamic—which have yet to be researched. Applications that require safe wireless sensor networks such as the Internet of Things, critical e-health systems, and ambient intelligence cannot be successfully addressed without these kind of tools.

So-called monitoring systems—or simply “monitors”—are used to evaluate the performance and operation of a sensor network in controlled conditions or even in a real environment. Monitors can focus on many performance parameters, such as throughput, jitter, response time or reliability, and even on security and intrusion detection in the network, as described in [4].

The use of these monitoring systems could be helpful in all stages in the life-cycle of a WSN. WSN researchers could use a monitor to perform comparative analysis of new proposals. Designers could use a monitor to select the best suitable techniques for a given application’s requirements. In implementation, the enhanced debugging capabilities brought by these monitor tools are unbeatable. Deployment is much easier when the correct functioning of the nodes can be verified *in situ*. During operation, malfunctions could be diagnosed without stopping the system and any system redesign could benefit from more detailed information about current functioning. In addition, these tools could become fundamental in the standardization and certification of applications based on WSN. Although monitoring systems generate a non-negligible cost, they are necessary to detect problems in the deployment of the wireless solution. These costs, as with many measurement tools, may be considered to be justified as far as the monitoring system is only attached when necessary, is used for a finite amount of time and then may be removed to be reused, if possible, in another monitoring activity.

Monitors are usually built following one of two possible approaches. Active monitors involve additional hardware and/or software in the sensor nodes, interacting with them. Consequently, active monitors usually require the modification of the sensor nodes to be monitored. This interferes with the node’s normal operation and measured parameters may vary from those of an unmonitored node. However, more variables may be observed, and thus the data obtained are more accurate.

On the other hand, passive monitors rely on the observation of the external behavior of the monitored system without any interference with its normal operation. Machine-learning algorithms [3]—which analyze the behavior of the system—may be used to evaluate and predict the presence of errors, undesirable operation or unexpected events. The monitor does not interfere with the monitored nodes, but only externally observable variables can be measured.

There is also another approach for monitor construction, which depends on whether the monitor is based on hardware or software. A software monitor is implemented by means of a specific code, application, or plug-in to the operating system of the node, which accesses the system status and reports relevant information. Usually, a software monitor yields in-depth information about the system’s performance, but it may interfere with the operation of the monitored system.

A hardware monitor consists of electronic devices connected to the monitored system, which collect data from interesting system points. Hardware monitors are usually less intrusive than software monitors, but they involve the use of additional components.

Each monitor approach by itself cannot cover all aspects of monitoring tasks, as we will discuss in the next section. Monitors can also combine both approaches (hardware and software) in order to achieve the advantages of both types and obtain a complete vision of the system, while trying to keep interference to a minimum. These are the so-called hybrid monitors [5].

This paper presents an active low intrusion hybrid monitor [6], based on both hardware and software. This monitor can record the events which occur in a node of a sensor network and store them in a non-volatile memory for later analysis. Moreover, it can be incorporated into a complete monitoring platform which includes other acquisition possibilities, such as passive monitors, as described in [7].

This paper is structured as follows: after the introduction, monitoring tools are described in Section 2. Section 3 explains the architecture of the non-intrusive hybrid monitor. The implementation of the monitor is described in Section 4. Section 5 details the evaluation of the intrusion produced by the monitoring tool. Finally, a discussion and conclusions are presented in Section 6 and Section 7.

## 2. Monitoring Tools

There are several monitoring sensor networks tools and techniques; most of them follow one approach and focus on WSN. In [3], both monitoring and debugging tools are considered and compared. This section contains a brief summary of some of most important and relevant tools.

SNMS (Sensor Network Management System) [8] and Sympathy [9] are two of the earliest and best-known monitoring systems. SNMS is a complete management system, focused on working with any type of sensor network. It is built on TinyOS [10]—an open source operating system designed for low-power devices—and enables a review of the state of a node and information to be saved locally. Nevertheless, it generates substantial intrusion and is oriented to management rather than monitoring.

On the other hand, Sympathy works as a passive monitor, and can detect and debug pre-and-post deployment errors. It operates by analyzing the data arriving at the sink of a sensor network, applying metrics, and inferring where in the network a fault or failure may occur. It also considers the aggregation of a small overhead in the network to increase its accuracy. However, it only considers the transmitted data and thus cannot observe the internal node information, something which could increase monitoring accuracy.

SNIF (Sensor Network Inspection Framework) [11], Pimoto [12], LiveNet [13], SNDS (Sensor Network Distributed Sniffer) [14], NSSN (Network monitoring and packet Sniffing tool for wireless Sensor Networks) [15], and EPMOSt (Energy-efficient Passive Monitoring SysTem for WSN) [16] are examples of passive monitors. Their approach consists in deploying a network of sniffers with an interface to capture all transmissions from nodes. The main difference between them is how the captured data is processed. Some of them transmit the data—via TCP/IP (Transport Control Protocol/Internet Protocol) or another radio interface—to another device for processing, and others can function as a sink, collecting and analyzing the information. They can also provide real-time analysis of data sensor network operation. Nevertheless, all these tools only capture the transmitted frames “on the air”; they cannot obtain information directly from nodes.

Despite its name, Passive Diagnosis (PAD) for WSN [17] is an active monitor system with low intrusion. It is based on a probabilistic diagnosis approach—based on a Belief (or Bayesian) Network—to infer the root causes of abnormal WSN operation. This adds a probe in each node which marks the packets with relevant data with very little overhead. However, PAD has to wait for a message transmission to send information and it might not determine when an error has occurred. Moreover, as non-sense nodes (such as router nodes) do not send sensed data, they are unable to send any monitor information to indicate possible abnormal operation.

Memento [18] and Lightweight Tracing [19] are examples of active monitors. Both use short encoding with sensor node events and information. The first adds its code protocol to a message and transmits it. Memento can detect problems in a node by using information provided by their neighbors in the network. In Lightweight Tracing the events are stored in non-volatile memory by using a very light coding. Further reconstruction and debugging of node and network behavior is then possible. Because both are active monitors, they generate substantial intrusion.

Minerva [20] is not a monitor, but a test-bed for WSN. It uses a debugging port and tracing port connected to the sensor node to observe the behavior of the node. Minerva has very interesting features but is inadequate for monitoring in real environments.

Finally, Spi-Snooper [21] integrates hardware and software in a hybrid approach. The hardware architecture brings the sensor node and the monitor together in a single unit in a transparent manner. The monitor spies on the SPI interface used to connect the sensor node to its radio module. The software architecture has two operation modes: active and passive. In passive mode the monitor—called the co-processor—mainly logs the communication through the SPI bus and checks some node data. In active mode it assumes the control of the SPI and the radio interface. However, it can only be used in sensor nodes that transmit through a SPI port. Only the data transmitted through this SPI interface can be monitored. Obviously, this technique cannot be used in sensor nodes with built-in radio modules.

Each one of these proposals has advantages and disadvantages. The proposed active monitors usually involve a great deal of intrusion. Meanwhile, the proposed passive monitors can only observe transmitted data, they are unable to observe events inside the node. Both the addition of monitoring information to a transmitted message and new messages to the network cause a decrease in network performance. Monitors based exclusively on software cannot work if the node fails. Hardware-based proposed monitors are too architecture-specific. Consequently, a monitor system with sufficiently broad network information coverage which also keeps intrusion low is required. Furthermore, this system has to be sufficiently generic to be applied to all hardware architecture. Many characteristics of the proposals studied were taken into consideration to form a base for the design of our monitoring system, which also attempts to minimize the drawbacks of these previous tools.

## 3. Architecture of a WSN Monitor

### 3.1. Specifications and Characteristics of the Monitor

Taking into consideration the findings from many previous deployments of WSN, the characteristics of wireless sensor network monitors (addressed in Section 1) and the existing proposals discussed in Section 2, the following specifications are deemed necessary for a new WSN monitor:

1. It must be able to observe all the relevant data. This would endow it with significant debugging potential.
2. It must be as hardware/software independent as possible.
3. It must be reusable and configurable.
4. The monitor must be easily attachable and removable, without stopping the operation of the monitored system.
5. The monitor must cause minimum disturbance to the operation of the observed system (low intrusion).

When addressing these specifications, software traps are considered to be the best way to achieve a high observability while maintaining high flexibility, as the designer defines the relevant events and introduces the trap code in the required places in the code. In order to achieve a very low intrusion level, a hardware attachable node frees the monitored system from the task of monitoring data management. The communication between both nodes must be performed through standard interfaces already available in most of the monitored nodes, providing reusability and hardware independence. Consequently, an active hybrid hardware/software solution is considered to be the best proposal.

Moreover, although monitoring systems are usually expensive, this approach minimizes costs because the proposed system is reusable and scalable, adjusting to monitoring requirements.

Finally, it is particularly interesting that the design of the monitor is based on a modular approach to make the system easily adaptable to new monitoring environments and improve reutilization. In this way, the design must be partitioned following a hierarchical layered architecture in order to attain modularity, making it possible to change monitor modules without changing the whole implementation.

### 3.2. Proposed Architecture

In this section the architecture and operation of the proposed monitor is presented. It is based in a standard-oriented architecture in order to provide benefits such as universality, reusability and flexibility.

As observed in the bibliography and discussed above, every monitoring system has to address some issues in order to be functional. Our proposal identifies these problems and classifies them into three categories:

Monitoring data. These data must be captured, analyzed, and shown to the user in a meaningful way. The semantic meaning of the data depends on the application and the monitoring requirements.

Once obtained, data must be presented in a standard format to ensure the integration of data provided by heterogeneous sources. Some issues related to this category include a common time base and capturing conditions.

As the monitored system is distributed in space, all the obtained data must be centralized and stored to provide a global comprehension of the whole system functioning.

**Figure 1:**
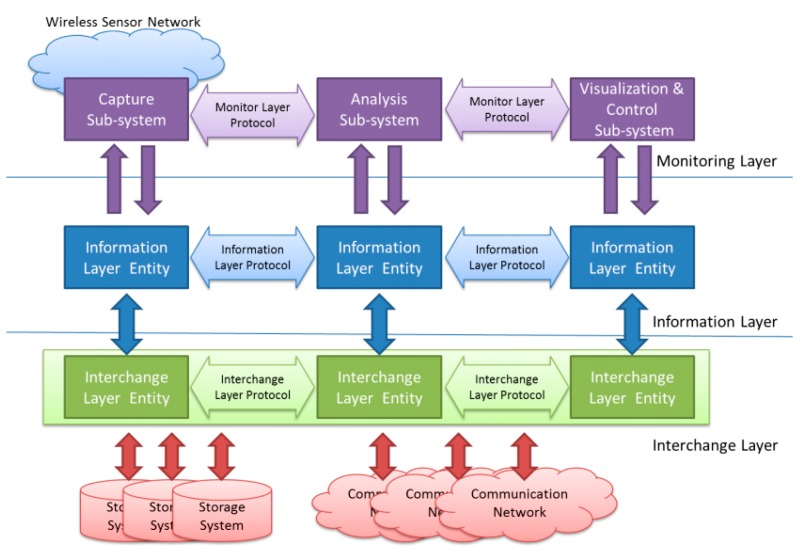
Architecture of the proposed hybrid monitor.

Keeping these different—and usually independent—problems in mind, the proposed architecture is composed of three layers, as shown in Figure 1.

The Monitoring Layer will be located in the upper level of the architecture. This layer is in charge of all the issues related, and specific to the WSN under observation. It must deal with the definition of what should be monitored, how this information should be acquired and the way it has to be processed and shown to the user.

The Information Layer is located under the Monitoring Layer. It must represent the obtained information in a standard way. This level also deals with timing issues, such as when the information must be captured (triggering) and storing this time value with the obtained data (time stamp).

Finally, the Interchange Layer allows the information captured at different points alongside the monitored WSN to be transferred and stored. The upper layers will retrieve this information to be analyzed and/or visualized by the Monitoring Layer.

In this three-layer architecture the communication between layers and entities in the same layer is similar to that standardized in the OSI (Open Systems Interconnection) reference model for networks [22]. Each layer defines interfaces to communicate with the upper and lower ones. One of the advantages of working with a defined architecture is that changes and developments in an entity/layer should not affect the other layers. Hence, any improvements to the monitor system will be easier to develop and implement. Moreover, it is straightforward to reuse this hybrid monitor in another sensor network by making the required changes in the respective layer.

## 4. The Active Low Intrusion Hybrid Monitor

In this section an implementation based on this layered architecture is presented. As the main objective of this implementation is to study intrusion, the implemented system is focused on all modules that affect said intrusion (capture subsystem, information layer, and interchange layer; first column in Figure 1), simplifying the other modules that do not.

### 4.1. Active Low Intrusion Hybrid Monitor Structure

**Figure 2:**
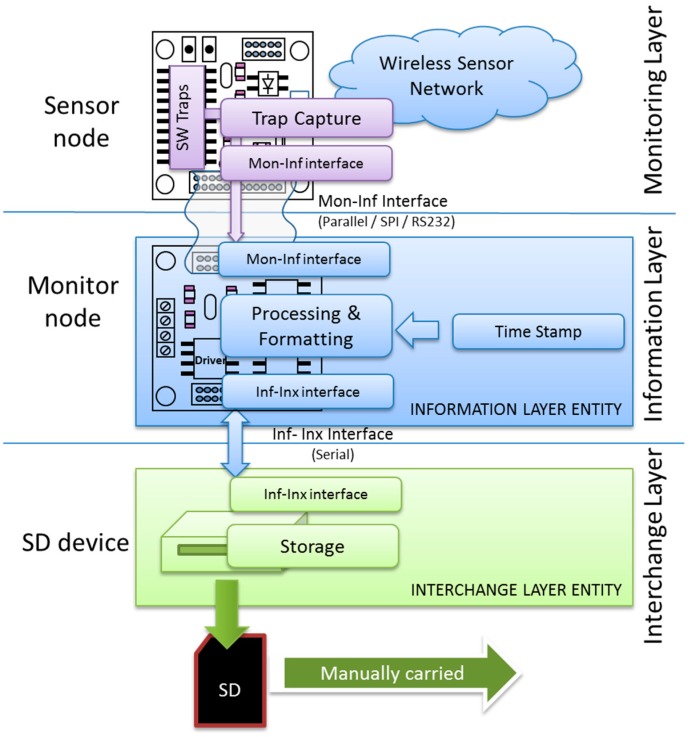
Implementation of the proposed hybrid monitor.

Figure 2 shows the structure of the implemented hybrid monitor. Three hardware devices can be identified: the sensor node to be monitored (mote), the attachable and reusable monitor node, and a storage device.

The capture subsystem in the Monitoring Layer is implemented by means of a software traps mechanism which runs in the sensor node hardware.

The Information Layer entity of the hybrid monitor is implemented in the monitor node. The interface between the sensor node and the monitor node, supporting communication between the Monitoring and Information Layers, is performed through a standard communications interface (serial, SPI, parallel, *etc.*). The use of these standard interfaces provides hardware independence and facilitates reutilization, making the attach/detach operation straightforward.

Finally, the Interchange Layer entity is implemented by means of a storage device handled by the monitor node, where the collected data will be stored. The communication between the storage and monitor node is done through a serial interface, as shown in Figure 2. Future implementations could consider the use of more sophisticated storage and distribution systems, such as distributed databases and secondary communication networks.

More powerful analysis and visualization subsystems, with lower layer support entities, are not considered at this point because they would not modify the intrusion evaluation carried in this research.

### 4.2. Monitor Operation

In order to observe and evaluate the behavior of the system under observation, the monitor must register the sequence of events that may characterize said behavior. Consequently, the first step must be to define the monitoring points—called probes—which are of interest to the designer.

With this information, the application code must be modified in order to generate the appropriate monitoring events. This modification is implemented using software trap mechanisms.

When a trap is fired, some associated information bytes are generated that may include additional information along with the event code. This monitor allows the designer to decide which additional data can be included, so providing a highly flexible monitoring tool, and increasing the accuracy of obtained information. For example, an error event may include an additional error code that better describes its causes. A transmission event may include part or the whole message, making a packet traceable through the sensor network. This can be considered a significant improvement when compared with other proposed systems that record events but cannot associate additional information to them.

These bytes must be processed, formatted, and recorded in a log file. Most active monitors require the observed node to run these processes. Our approach frees the observed sensor node from having to run said processes—and thus reduces the intrusion—by means of the attached monitor node. The monitor node is in charge of these processes, which include time stamp, data format, data storage or (in future developments) data transmission.

**Figure 3:**
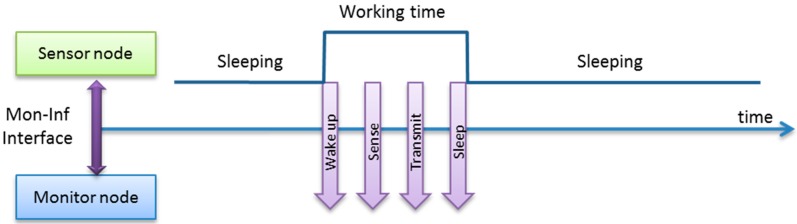
Hybrid monitor operation outline.

Our solution (Figure 3) only requires the trap capture routine to transmit the event and its associated information to the monitor node through the Monitoring-Information interface (Mon-Inf interface). The monitor node is in charge of processing the data, including time-stamping. It also applies a standard data format, as described below. Finally, the monitor node must also record this data. In this approach, the data is stored in a SD (Secure Digital flash memory) module through the Information-Interchange interface, implemented by a serial link.

In order to minimize the intrusion, the time and resources required for the sensor node to send the monitoring messages must be as low as possible. One of the main advantages of using standard interfaces is that most sensor nodes include specific hardware to perform the transmission in parallel with normal microcontroller operation, without disturbing it. The degree of parallelism obtained between both operations is critical in determining the level of intrusion. That is the reason why this article evaluates the intrusion obtained when the most commonly-available interfaces are used for this Mon-Inf communication.

**Figure 4:**
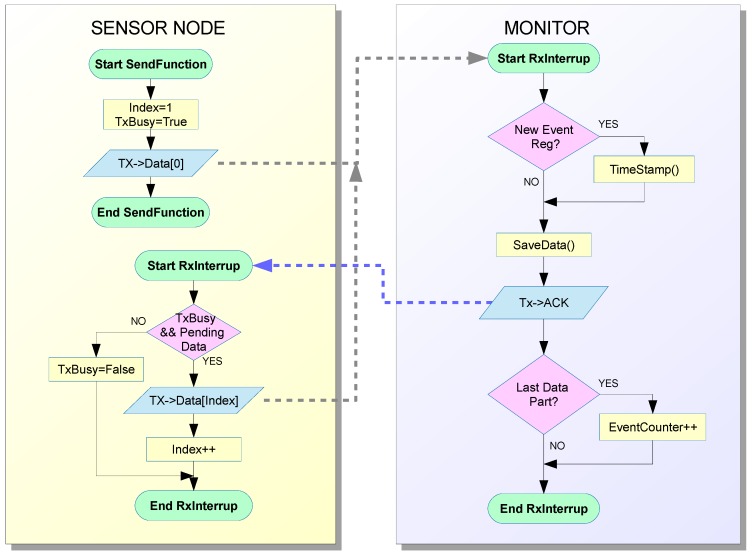
Flowchart of hybrid monitor operation.

Figure 4 shows the data flow and operation of the implemented hybrid monitor. The tasks which are usually performed by active monitors in the observed node have been divided between the sensor node and monitor node. The operations to be performed by the sensor node have been minimized and the rest, such as time stamp, data format, data storage, *etc*. have been moved to the monitor node. Communication routines have been included in the sensor node to transmit the data.

Consequently, as shown in Figure 4, two routines are included in the Monitoring Layer inside the sensor node. The first of them—which can be called as a routine by the code running in the sensor node—prepares the data about events to be sent to the monitor and sends the first bytes. As the monitor can manage traps that require many bytes, the message may be too long to be transmitted in just one iteration. In this case, a second routine is activated by an interruption when an ACK (acknowledgment message) is received from the monitor node. This routine sends the additional data until the end of the message.

The use of an ACK mechanism has experimentally proved to be necessary to guarantee a more reliable monitoring operation. The intrusion introduced by this ACK mechanism can be considered very low, due to the low priority interrupts used in implementation, as discussed below.

The monitor node is always waiting for information from the sensor node. This data must be processed when it is received, which involves several operations. Data must be stamped with the appropriate time stamp. When the first byte arrives, the time stamp is registered. Then, the monitor node receives the rest of the message. An ACK message is sent to the sensor node for every piece of data received. The message has to be processed when all of it has been received. In this implementation, the message treatment consists in the creation of a record which includes the date, time stamp, event code, and additional data (which is used in our example to indicate the iteration number).

This implementation uses a 4-bit coding for events, as shown in Table 1. Designers are free to define their own codes (and additional data) in order to adjust the system to the monitoring and application operation they require.

Finally, after the monitoring campaign, monitor nodes should be removed to be reused in other monitoring campaigns, even on WSNs whose sensor nodes are based on different hardware architectures.

**Table 1:** Example of 4-bit event codes.

Code	Meaning
#define Log_Reset 0x00	//Node Reset/Initialization
#define Log_Sense0 0x01	//Read sensor 0 (first/unique)
#define Log_Sense1 0x02	//Read sensor 1 (second, if it exists)
#define Log_Sense2 0x03	//Read sensor 2 (third, if it exists)
#define Log_Wakeup 0x04	//Wake up from sleep/stop
#define Log_RxData 0x05	//Node receives data
#define Log_TxData 0x06	//Node sends data
#define Log_RxACK 0x07	//Node receives ACK
#define Log_RRoute 0x08	//Node reroutes data (if applicable)
#define Log_Sleep 0x09	//Node goes to sleep mode
#define Log_Stop 0x0A	//Node goes to stop mode
#define Log_LowBat 0x0B	//Low battery indication
#define Log_SinkRx 0x0C	//Sink receives data
#define Log_SinkTx 0x0D	//Sink sends data
#define Log_SinkEr 0x0E	//Error in sink
#define Log_Error 0x0F	//Error in node

### 4.3. Hardware Implementation

**Figure 5:**
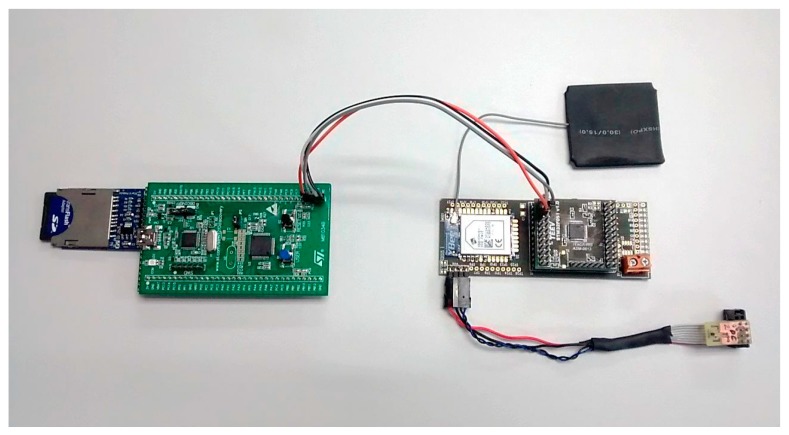
Monitor node connected to sensor node.

Figure 5 shows the hardware implementation of the monitor shown in Figure 2. The monitor node (on the left) is connected to the sensor node (on the right). The monitor node has been implemented using a commercially available microcontroller system, based on the STM32F051R8 ARM Cortex-M0 microcontroller (STMicroelectronics, Geneva, Italy). The authors consider this architecture to be representative in current applications. This microcontroller also offers several common interfaces—GPIO (General Purpose Input/Output), SPI, USART, and others—which can be used for our purpose [23]. The monitor board has been connected to a SD card through its SPI interface.

The implementation costs in the sensor nodes is, usually, very low. Commonly, microcontrollers in sensor nodes have free communication interfaces that can be used to send the traps to the Monitor node. The only additional hardware engineering required is that the selected interface is externally available through a connector.

Figure 5 also shows a sensor node, which is used for monitor evaluation. It is a previously used sensor node which has already been deployed in a real temperature control application. It is based on an ARM Cortex-M0 (STMicroelectronics, Geneva, Italy) and includes a XBEE (Digi international Inc., Minnetonka, MN, USA) wireless communication module, a MAXIM LM75 temperature transducer (Maxim Integrated Products, Inc., Sunnyvale, CA, USA) and an external antenna. It runs an application that periodically wakes up from sleep mode, takes a measurement from the temperature sensor, transmits the results through the wireless communications module, and enters into sleep mode for 60 s. In this sensor node no hardware modifications were required, as the three studied interfaces (SPI, GPIO, and USART) were available through the connectors.

The intrusion caused by the active hybrid monitor is related to the communications interface between the sensor node and monitor node. To evaluate this intrusion, three different interfaces were considered. The interfaces studied, which are usually found in most microcontrollers, were SPI (shown in Figure 5), USART (Universal Synchronous Asynchronous Receiver-Transmitter), and parallel transmission using 16 GPIO ports. Parallel and SPI data width is 16 bits, and USART data width is 8 bits. Moreover, three transmission speeds were evaluated for the SPI interface. To compare the performance of each interface, four sizes of transmitted data (common sizes of 16, 32, 64, and 128 bits per trap) were used. The larger the data being transmitted, the more detailed the information provided in each trap, but also the higher the interference.

The monitor node constitutes the Information Layer entity of the monitor. It has a built-in RTC (Real Time Clock), which is used to generate timestamps for the event register.

### 4.4. Software Implementation

The software part of the presented active hybrid monitor consists in a library added to the sensor node and the code in the monitor node.

The library on the sensor node offers an Application Programming Interface (API) which allows the designer a user-friendly way to introduce monitoring capabilities into its application code. This has been implemented using software traps and constitutes the capture subsystem of the Monitoring Layer. These software traps consist in a set of instructions located in the code of the monitored node. They send information when they are executed, usually related to significant operations—from a monitoring point of view—in the node, and can also send optional additional information, such as the value of a variable or the contents of a message.

Table 1 shows an example of a software trap used in the presented hybrid monitor. The code shown uses parallel communication as the interface between both nodes. The WriteLog function in the main procedure is a software trap which has been included in the application code. The parameters trap code (Log_sense0) and measured value (temper) are intended to be recorded by the monitoring system, indicating a temperature has been reached and its current value.

The implementation of the WriteLog function is in charge of transmitting the value through the desired interface. In this example, only 16-bit-length traps are considered and the acknowledge mechanism is omitted for simplicity.

As can be seen in Table 2, software traps are implemented as a routine call with very few instructions and thus cause very little intrusion. The monitoring layer also considers the trap collection procedure in the sensor node, as shown in Figure 2, which must transmit the trap data through the standard communication interface. The left block in Figure 4 shows this transmission flowchart in the sensor node. The right block in this figure shows how the monitor node implements the software required to receive this data.

**Table 2:** Sample of software trap program code for parallel interface.

int main (void){ // application code
…
TempValue = LM75_ReadTemp(0x94);
TempCels = TempValue >> 4;
temper = TempCels * 0.0625;
**WriteLog(Log_Sense0,(uint8_t) temper); // Software Trap**
SendValue(temper);
…
} // end application code
void WriteLog (uint8_t cod, uint8_t val){
uint16_t buff = cod | (val<<8); //Prepare buffer = code + value
GPIO_Write(GPIOB, buff); // put buffer through parallel port
GPIO_WriteBit(GPIOA, GPIO_Pin_0, Bit_SET); // Write pulse (up)
GPIO_WriteBit(GPIOA, GPIO_Pin_0, Bit_RESET); // Write pulse (down)
}

Both the sensor node and monitor node may benefit from the availability of standard communication libraries, providing independence between hardware and software. For instance, Cortex Microcontroller Software Interface Standard (CMSIS) has been used in both sides to implement these routines. This provides a standardized level, which may be easily reused in other CMSIS based microcontrollers and even ported to other architectures applying this methodology to their own libraries [24]. As the messages sent through the interface between the sensor node and monitor node do not change, the monitor node requires no modification.

The monitor node also implements the Information Layer, as shown in Figure 2. Consequently, it is in charge of applying a time stamp and data formatting to the received traps.

The monitor node is also responsible for Interchange Layer functions. Figure 2 shows that this implementation of the Interchange Layer consists of a non-volatile memory (Secure Digital—SD—card) where the captured information will be stored. This approach is similar to that used in Lightweight Tracing [19], but in our proposal this memory is attached to the monitor node instead of the sensor node. Future versions of the Interchange Layer could include a radio interface to transmit the collected information, either in real time or in a scheduled manner.

### 4.5. Monitoring Data Obtained

Although the main objective of this paper is not the application of the monitor to a real sensor network, but to study of the intrusion caused by it, the validation of the correct behavior of the monitor was considered to be a necessary, previous condition.

**Figure 6:**
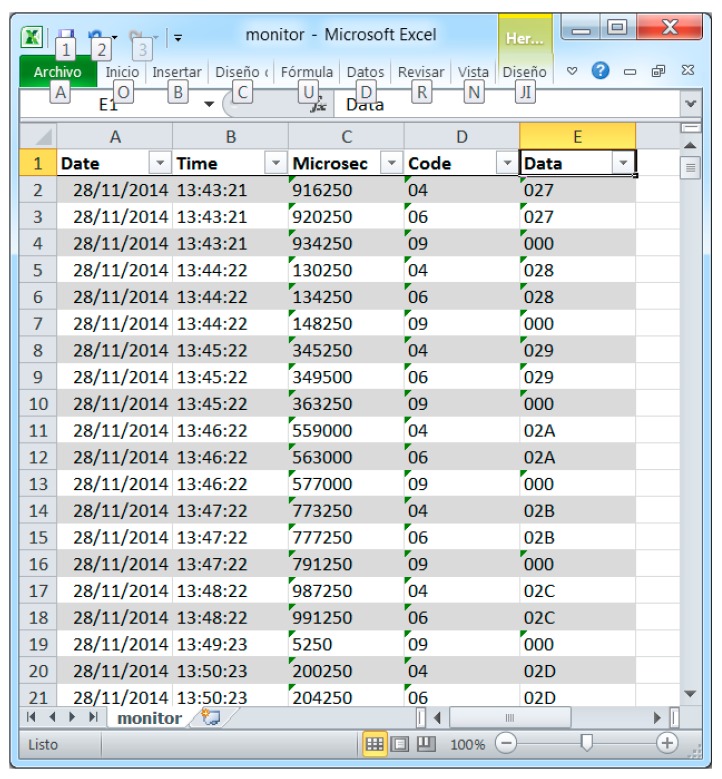
Information recovered from the SD card attached to the monitor node.

Figure 6 shows a trace of the information registered by the monitor in a single node. Three kinds of events—wake up, transmission, and sleep—were deemed sufficient to measure the intrusion and, thus, only three traps were inserted in the code of the sensor node to be monitored. These codes are sent to the monitor node through the Mon-Inf interface. The monitor node processes the data in the Information Layer, adding the time stamp information and formatting said data in CSV (Comma Separated Values format (ASCII—American Standard Code for Information Interchange—text separated by commas). Finally, the Interchange Layer is also implemented in the monitor node to store said data in a SD card.

CSV format was selected for the Information Layer because of its portability, it being compatible with most analysis and visualization tools. These tools are sufficient for the evaluation of the intrusion (the main objective of this research). Future implementations will improve the functions offered in analysis and visualization subsystems in accordance with the architecture shown in Figure 1.

Figure 6 shows part of the information generated by the hybrid monitor after recovering the produced file from the SD card. This information includes date, time, ms, event code, and additional data. Event codes 04, 06, and 09 (Table 2) have been used for wake up, transmission, and sleep events, respectively. Additional values for wake up and transmission events—also sent as additional information—indicate the iteration number. All this data can be delivered to the visualization and control subsystem (Figure 1) by using the Information Layer services.

## 5. Intrusion Evaluation

Three principal intrusion aspects must be considered. Time intrusion deals with the increase in execution time of the sensor node caused by the monitor. Taking into account that sensor nodes usually have a limited flash memory, code intrusion evaluates how many additional bytes are required to implement the monitoring of the sensor node’s program. Finally, energy intrusion evaluates the additional energy the monitoring operation requires. Many experiments were performed, following a detailed experimental plan, which took into consideration the previously cited interfaces and data sizes in order to measure the intrusion of the active hybrid monitor. The results obtained are presented in this section.

In order to obtain reliable results, *n* replications were performed for each measurement, *n* being calculated as follows:

The results for each measurement were considered as random variables (*X*_1_, *X*_2_, …, *X*_n_) with a μ mean value. Measurements were repeated n times until an estimation of μ was obtained with a 90% confidence interval according to Equation 1, where t_n−1,0.95_ represents the upper limit of the Student’s t-distribution on n − 1 degrees of freedom, and *X*(*n*) and *S*^2^(*n*) are the mean and the variance of the results obtained in the different experiments:

(1) X¯(n)±tn−1,0.95S2(n)n

### 5.1. Time Intrusion Analysis

Time intrusion was determined by the replication of an experiment which consisted in measuring the amount of time needed to fulfill one thousand application iterations in the sensor node during both monitored and not-monitored operation. During monitored operation, a single software trap was added to be fired when the wake-up event occurred. The time required to fulfill the iterations in monitored mode is called monitor mode execution time. The execution time of the same program in the sensor node without traps was also measured (hereafter referred to as reference time). The program consists of a wake-up, measurement of the value from a transducer, and pass-to-sleep mode operations, repeated one thousand times. Reference time was found to be 5805 ms.

As previously mentioned, three interface implementations and four data sizes were combined to yield the results in Table 3. This table shows the difference between the monitor mode execution time and the reference time, divided by the number of iterations. As there is one trap per iteration, this value is the time intrusion per trap.

**Table 3:** Time intrusion in sensor node for each log event (ms).

Data Size	Parallel (16 Bits)	SPI 18 mbps	SPI 4.5 mbps	SPI 2.25 mbps	USART 115.2 kbps
16 bits	3.80	3.10	3.10	3.20	6.20
32 bits	7.30	6.10	6.20	6.20	13.60
64 bits	16.50	13.00	13.10	13.10	28.80
128 bits	31.90	28.10	28.10	28.20	60.60

SPI and USART interfaces with dedicated hardware allow the communication processes to be executed concurrently with application code execution. In these interfaces (see code in Table 1), trap routines are not concerned with communication and they merely write the outgoing data into the interface buffers. Then the sensor node program may continue. On the other hand, the parallel interface has no dedicated hardware controller and an additional line is required to generate a reception interruption in the monitor node. That is the reason why the time intrusion is slightly greater than when using the SPI interface. As expected, time intrusion increases for larger data sizes. In the case of USART the intrusion time is about double that of the other interfaces, because it only sends 8 bits per transmission (the other interfaces considered send 16 bits) and it has to manage twice the number of interruptions.

**Figure 7:**
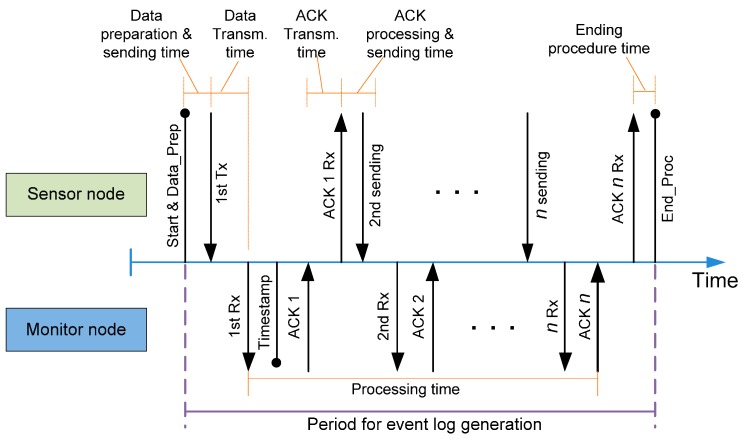
Time outline for event log generation.

Figure 7 shows that the time required to process a trap event in both the sensor node and monitor node. Despite the fact that the sensor node can execute part of its application code while the trap is being processed, it is not possible to launch a new trap before its treatment is complete. This minimum time between traps is shown in Figure 7 as the “Period for event log generation”.

This period starts when the sensor node code reaches a software trap. The trap code and its associated data must be prepared to be sent in several messages through the Mon-Inf interface. When it is ready, the dedicated hardware in the sensor node is in charge of transmitting the first message containing this data to the monitor node. After that, the monitor node generates the time stamp and acknowledges the transmission to the sensor node. As the ACK is received, the sensor node transmits the next message containing the trap information. This process repeats until all the messages related to the trap event have been transmitted.

The time intrusion (Table 3) every trap introduces is the sum of several time intervals (red boxes in Figure 7).

It can be seen that this time intrusion in the sensor node is not affected by the communication time. The hardware implementation in modern microcontrollers allows the CPU to execute code (application code) while transmission is being performed. However, this communication time is not negligible when dealing with the maximum event generation rate, and it has to be taken into consideration to evaluate the maximum frequency event generation rate.

This frequency is related to the processing time in the monitor node. This time has also been measured and Table 4 shows the values obtained. As shown in Figure 7, the processing time is measured from the arrival of the first data to the receipt of the last piece of information. Timestamp generation takes 15.8 ms and is performed when the first data message of the trap arrives.

**Table 4:** Processing time in monitor node for each event log (ms).

Data Size	Parallel (16 Bits)	SPI 18 mbps	SPI 4.5 mbps	SPI 2.25 mbps	USART 115.2 kbps
16 bits	17.80	17.40	17.40	17.40	88.40
32 bits	22.90	23.40	25.40	27.80	262.00
64 bits	32.90	34.40	40.10	48.20	612.00
128 bits	52.80	56.20	69.80	88.00	1300.00

As shown in Table 4, when dealing with 16-bit events the times obtained are similar for every interface—except the USART—as the process time depends mainly on timestamp generation. For larger data sizes the processing time is slightly less when using the parallel interface instead of SPI, especially when the SPI speed is low. In the case of the USART interface, the processing time is much greater because of its low transmission speed and the fact that it can only send 8 bits at a time, whereas the other options considered can send 16 bits at a time. As expected, the processing time increases for larger data sizes.

The values in Table 3 and Table 4 are dependent on the architecture used (48 MHz ARM Cortex-M0, STMicroelectronics, Geneva, Italy) in both the sensor node and monitor node. A monitor node based on a faster microcontroller will reduce the timestamp generation time, and hence will also reduce the processing time and increase performance. Moreover, the time intrusion and processing time could change depending on the sensor node characteristics, such as core frequency, buffer availability, architecture, interface to monitor, among others. Nevertheless, the time intrusion in the sensor node is very low when compared with the processing time in the monitor node. The relationship between both times is not architecture-dependent. This means a low degree of time intrusion can be expected when monitoring other sensor node architectures.

When comparing this proposal with others in the bibliography, it is important to note that active non-hybrid monitors make the sensor node perform all the monitoring tasks (data capture, formatting, and storage); meanwhile, with our proposal, the time needed to perform all the aforementioned tasks is replaced by a small communication time, thereby substantially reducing the intrusion.

A USART interface was not deemed a good option to be used in our monitor because of its comparatively low speed and high processing time which result in low performance. Therefore this interface is considered in further results for comparison purposes only.

### 5.2. Code Intrusion Analysis

As memory resources are limited for sensor nodes, the evaluation of the intrusion on program code is relevant. Code used in the sensor node was generated by Keil Microcontroller Development Kit (MDK) version 5, a comprehensive software development environment for Cortex-M processor-based microcontrollers which includes an Integrated Development Environment (IDE), Compiler, and Debugger [25].

Size difference in bytes between the pure application code and the trap-modified code has been considered in a binary compiled program. The main differences between both codes consist in the addition of the port initialization subroutine and several data transmission instructions. Table 5 shows the intrusion in bytes on program code in the sensor node. Intrusion is not related to the communication speed, thus this parameter has not been considered.

Since transmission code is reused for all the traps, a new event may be monitored by merely adding a trap call of 8 bytes to the sensor node code.

**Table 5:** Intrusion on code: Initialization and one event log (bytes).

Interface	16 Bits	32 Bits	64 Bits	128 Bits
Parallel	228	252	268	276
SPI	428	452	468	476
USART	292	316	332	340

Code intrusion in size (*S*_Intrusion_) may be predicted by means of Equation (2), where *S*_init_ is the value appearing in Table 5 (which corresponds to the initialization and interrupt routine), and *n* is the number of trap calls included in the application code. As expected, for a small number of monitored traps, the code intrusion is mostly determined by the initialization code.

(2) $$S_{Intrusion}=\;S_{init}+8\times \left(n-1\right)$$

### 5.3. Energy Intrusion Analysis

Power consumption is a key aspect of sensor network nodes. This is the reason for the increasing number of microcontroller systems which include specific hardware to monitor their own power consumption. Furthermore, some microcontrollers, such as STM32L0 (STMicroelectronics, Geneva, Italy), are able to measure their instantaneous power consumption without additional hardware. These power measurements may be used to handle the node energy efficiently, and may even be sent with the data to provide global energy management.

In this environment, a complete monitoring tool must be able to handle the relevant information about the energy behavior of the sensor node.

Due to energy restrictions in this kind of systems it is very important to reduce the energy consumed by the monitoring operation. Consequently, the best solution consists in the addition of an external self-powered node performing passive monitoring, which introduces no energy intrusion. Nevertheless, the solution proposed in this paper seems to be very close to these solutions, using an external monitor node but increasing observability by using active monitoring solutions (software traps). It must be noted that the monitor node include its own power source (battery, energy harvesting techniques or even a wired power installation) to avoid changing the behavior of the monitored sensor node.

The monitoring tool should be aware of the intrusion in power consumption that it introduces. Consequently, this section studies the energy intrusion caused in the sensor node when the monitoring system is capturing. This intrusion is caused, on the one hand, by the communication hardware interface used to support the Mon-Inf interface and, on the other hand, by the time taken to execute the trap. The latter has already been addressed in Section 5.1.

To evaluate the power consumption of the communication hardware interface with and without monitoring operation, the instantaneous power consumption in both cases has been measured by means of a set of experiments. They were performed in accordance with the plan described in Section 5. Power consumption was determined by modifying the application program used in the sensor node in the time intrusion evaluation to make it into an infinite loop, avoiding sleep mode and keeping the peripherals used enabled. With this program in execution, about 7000 samples of sensor node electrical power were taken—18 samples per second—and averaged. Measurements were taken with an Agilent 34405A Multimeter (Agilent Technologies, Inc., Santa Clara, CA, USA). This multimeter has a 5.5 digit resolution, with an accuracy of ±(0.05% of reading +0.015% of range) for our measuring conditions [26]. Both non-monitored and monitored operation measures were repeated until the confidence interval was over 90% according to Equation 1.

These experiments were run for all the interfaces considered (parallel, SPI, and USART) and for several communication speeds and data sizes. The results obtained show that electrical current with no monitoring operation was 22.53 mA. They also demonstrate that the evaluated communication speeds and data sizes do not affect the instantaneous power consumption. Table 6 shows the ratio between the electrical current required in monitored operation compared with non-monitoring operation when each interface is used.

**Table 6:** Current increment due to monitoring operation by interface.

Interface	Measured Current (mA)	Increase on Non-Monitored Operation
Parallel	22.74	0.92%
SPI	23.08	2.38%
USART	22.86	1.44%

The monitoring operation introduces, as previously stated, two causes of energy intrusion: the electrical current increased by the communication hardware and the overtime introduced by the execution of software trap’s code. As a consequence, energy intrusion may be calculated as shown in Equation (3):

(3) $$∆E=E_{m}-E_{r}=V\times I_{m}\times T_{m}-\;V\times I_{r}\times T_{r}$$

where *E_m_*, *I_m_* and *T_m_* are the energy, current and execution time when monitoring, and *E_r_*, *I_r_*, and *T_r_* are the same variables when no monitoring is being performed. *V* is the power voltage in all cases. From this expression it is easy to deduce Equation (4):

(4) $$\begin{array}{cc} ∆E=E_{m}-E_{r} & =V\times I_{m}\times T_{m}-\;V\times I_{r}\times T_{r}=V\times [I_{m}\times T_{m}-I_{r}\times T_{r}]\\ & =V\times [(I_{r}+∆I)\times T_{m}-I_{r}\times T_{r}]=V\times [I_{r}\times T_{m}+∆I\times T_{m}-I_{r}\times T_{r}]\\ & =V\times [I_{r}\times (T_{m}-T_{r})+∆I\times T_{m}]=V\times [I_{r}\times ∆T+∆I\times T_{m}]\\ & =V\times I_{r}\times ∆T+V\times ∆I\times T_{m}=W_{r}\times ∆T+∆W\times T_{m} \end{array}$$

It can be seen that, as previously mentioned, the energy increment is caused by two factors. The first is the execution time increment due to monitoring tasks, and the other is the power consumption increment caused by the hardware used for monitoring. With regard to the first of these factors, the proposed hybrid solution only requires trap capture and basic communication tasks. With regard to the second, the proposed solution only requires the use of the communications hardware interface.

Finally, the percentage of energy intrusion may be calculated using Equation (5):

(5) $$\frac{∆E}{E_{r}}=\frac{I_{r}\times \;∆T+∆I\times T_{m}}{I_{r}\times T_{r}}$$

As all these data are known, it is possible to use the values for current increments (Table 6) and time intrusion (Table 3) to obtain the energy intrusion for each interface. Table 7 shows the values obtained. The energy intrusion can be considered low in relation to the normal operation of the sensor node (below 3%). From Table 7 it can be seen that the power consumption intrusion increases with data size and is higher for the parallel interface (as expected) due to the number of lines involved in trap transmission.

**Table 7:** Energy Intrusion (%) per event.

Data Size	Parallel (16 Bits)	SPI 18 mbps	SPI 4.5 mbps	SPI 2.25 mbps	USART 115.2 kbps
16 bits	1.00%	2.50%	2.50%	2.50%	1.57%
32 bits	1.06%	2.55%	2.55%	2.55%	1.70%
64 bits	1.22%	2.67%	2.67%	2.67%	1.97%
128 bits	1.49%	2.94%	2.94%	2.94%	2.52%

It can be observed that when using the SPI interface transmission speed has very little influence on energy intrusion. The small amount of data transferred by trap makes speed almost irrelevant (Table 3), and no consumption difference has been found (Table 6).

It must be noted that these values correspond to the worst trap generation scenario, where a very small application code is executed (only the capture of a sensor). A lower trap generation rate would significantly reduce this energy intrusion.

## 6. Discussion

From the previous numerical evaluation it can be concluded that, as observed, the use of a parallel interface presents a small advantage from the point of view of code intrusion. The parallel interface presents a greater advantage in energy intrusion, despite producing greater time intrusion, because it produces a low electrical current increment. However, a drawback of the parallel interface is that it requires approximately 20 lines (depending on the implementation), which are not always available in sensor nodes. If the lines are available, physical attachment should be straightforward using a parallel connector.

Serial USART-based interfaces also introduce a small amount of code, as it is a very common and easy-to-use interface. Its energy intrusion is close to the parallel case, despite its low transmission speed. Its greatest drawback can be found in time intrusion, which is many times greater than that presented by the other interfaces studied (Table 3). The shorter word length and lower transmission speed makes communication with the monitor node very slow (Table 4). This implies that the number of events per second (trap frequency) could be limited by the capabilities of this interface.

Finally, SPI-based interfaces present a balanced behavior between the other two options. Although its code intrusion is greater than the other interfaces, it is only 150 bytes approximately. Additionally, this interface permits high trap generation frequency. Finally, physical attachment and detachment of monitor node should be straightforward, as the required lines (only four) are externally available, using a serial connector.

The intrusion when the proposed monitor uses the studied interfaces can be considered low in all of the three aspects mentioned (time, energy and code intrusion). The difference between the interfaces is not definitive; in some cases the choice of one of them may be conditioned by other circumstances, such as the available interfaces in the sensor nodes or application/designer restrictions. Even when no other interface is available, the serial USART-based interface may be an appropriate solution, accepting its drawbacks.

To summarize the previous studies, a ranking based on a preference index is shown in Table 8.

**Table 8:** Preference index of each sensor-monitor interface.

	Parallel (16 Bits)	SPI 18 mbps	SPI 4.5 mbps	SPI 2.25 mbps	USART 115.2 kbps
Time Intrusion	4	1	2	3	5
Code Intrusion	1	3	4	5	2
Energy Intrusion	1	3	4	5	2

### 6.1. A Real Application

Previous analyses were performed in a high stress environment, with a high trap generation rate, in order to study the worst case scenario. This section describes the measurement of the influence of these intrusion effects when applied to the monitoring of a real WSN application.

The monitored application consists in a temperature measuring system. It is formed by a set of nodes with a temperature transducer which periodically (every 2000 ms) wake up, capture the temperature from the transducer, send it to the gateway (without routing capabilities), and finally return to sleep mode. The working time of this application is approximately 200 ms. The code size is approximately 12 KB, and its required energy before monitoring was measured at 24.58 mA on average when in active mode (working time). Both parallel and SPI interfaces were available for monitoring purposes.

In order to monitor this application, four traps were defined, registering four events: Node Wake Up from Sleep (0x04), Read sensor 0 (0x01), Node Sends Data (0x06), and Node Goes to Sleep Mode (0x09), as defined in Table 2. All these traps send 128 bits of additional data.

In the real application the execution time was measured for SPI and parallel interfaces. The time increment was found to be 130 ms and 120 ms when parallel and SPI interfaces were used respectively. Thus, the time intrusion caused was 0.065% in parallel and 0.06% in SPI.

The evaluation of the code intrusion in the proposed real application is very simple. Parallel implementation requires the addition of 300 bytes (see Equation (2)) that represents a code intrusion of 2.44%. On the other hand, SPI required 500 bytes of code, producing an intrusion of 4.07%.

Finally, electrical power consumption was measured in the sensor node when monitoring was active with both interfaces. Instantaneous power consumption when using the SPI interface was found to be 25.2 mA, and the parallel interface produced a measurement of 24.85 mA.

This increase of instantaneous power consumption, together with the additional time intrusion, produces an energy intrusion of approximately 2.6% in the case of the SPI and 1.2% in the case of the parallel interface. These results correspond to a four-event monitoring, so they are lower than those presented in Table 7 (four to five times lower). As has been said in the previous section, the results shown in Table 7 correspond to a worst-case scenario for trap generation rate.

### 6.2. Comparison with Bibliography Cases

**Figure 8:**
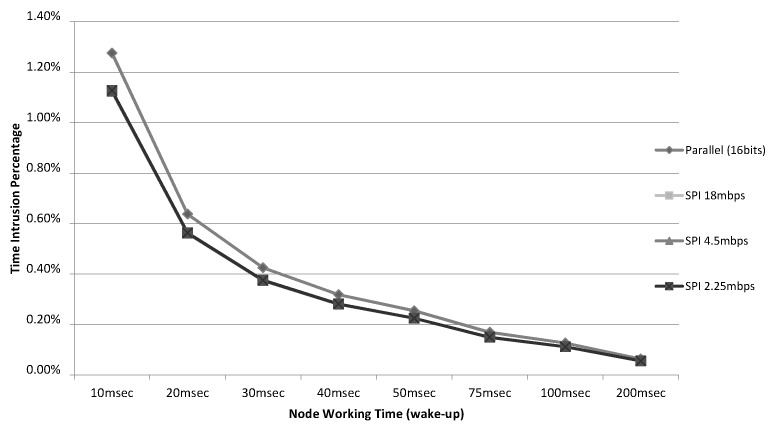
Percentages of time intrusion (128 bits/4 event logs).

The results are quite similar for both interfaces. In order to highlight the difference between them, Figure 8 shows how this intrusion would affect the working time values used in other real implementations found in the literature. For example, in [27] (a wireless sensor node to monitor track bicycle performance) the sensor node active time is 30 ms. In others cases, such as [28] (an application for heating and cooling loads) and [29] (a wireless node enabled by wireless power with some sensors) the node active time reaches 100 ms or more. In all cases, the percentage of intrusion is very low—less than 1.5% in the worst case—even in the smallest case (10 ms).

The time intrusion of active monitoring tools is much greater that those caused by the presented solution. For example, an active tool like Envirolog [3,30], in which the sensor node is in charge of capturing the trap events, processing and storing them in flash memory, can generate an overhead of 70% for 15 traps. This overhead seems unacceptable in a real scenario, explaining the interest in hybrid solutions to free the sensor node from most of the monitoring functions.

The overhead of instruction code (inside the sensor node) of active monitoring tools is higher than that associated with the hybrid monitor presented. For example, additional features of Sympathy occupy 1558 bytes of ROM (Read-Only Memory); EnviroLog requires 15,160 bytes of node flash memory [3]; and the Lightweight Tracing program’s requirement can reach approximately 4000 bytes [19].

No energy intrusion data was found in the bibliography studied. As the proposed systems consider the monitoring as a temporal phase inside the life time of a WSN, the energy consumption has not been analyzed in depth. However, active monitoring techniques require the monitored node to perform many operations to record its monitoring information. From Equation 3, it seems obvious that these techniques increase the power consumption as they increase the working time and, in most cases, they also require the use of hardware resources from the sensor node. Both increased working time and use of hardware resources result in an energy consumption many times greater than that observed when using the hybrid solution approach presented in this paper.

## 7. Conclusions

In this paper an active hybrid (software and hardware) monitor has been presented. It is able to collect detailed information about the operation of a sensor node with very low intrusion, and thus without affecting the node’s performance.

The software traps implementation is easily portable to many hardware platforms, as it has been released with open-access license. The libraries are intended to be freely distributed in order to be used without any royalties. Software traps become non-operational when no monitor node is attached, with a negligible impact on sensor node’s performance.

The design of the monitor node makes it easily attachable and detachable from sensor nodes. Monitor nodes may then be removed when the monitoring campaigns ends.

A monitoring campaign may be repeated at any moment, just plugging again the monitor nodes, if software traps are implemented. For instance, the monitor nodes may be used for debugging purposes at implementation stage, then removed—just disconnected—when sensor nodes are finished. Later, after the deployment of the WSN, if undesirable behavior of sensor nodes is detected, monitor nodes may be reconnected again to evaluate the *in situ* operation. When the problem is detected and corrected, monitor nodes may be removed again.

At the same time, monitor nodes use standard interfaces in order to be attachable to many different sensor node architectures. The monitoring tools found in bibliography are always hardware-dependent, being built for a specific sensor node.

The monitor nodes also include its own power source, battery based or even wired power installation, to avoid changing the behavior of the monitored sensor node.

As a first step, and following a study of monitoring tool requirements, a new architecture was presented for monitoring systems. This architecture addresses all the characteristics and functions required in monitoring tools, structured in a hierarchical way. It also provides advantages such as flexibility, reusability, and standardization.

Using this architecture, an active hybrid monitor was implemented. It is based on the use of new additional hardware, called a monitor node, which must be attached to the sensor node that is to be monitored. This monitor node is in charge of many monitoring functions—data format, data storage, time stamp, *etc.*—reducing the interference to the sensor node application.

The monitor node is complemented with software which is introduced into the sensor node. This piece of software captures the relevant events to be monitored and uses a standard interface to transmit them to the monitor node if present. The designer may define their own relevant events to monitor, and introduce them into the application code by means of software traps. This mechanism offers both flexibility and the possibility of obtaining detailed data regarding the internal behavior of the sensor node. If no monitor node is attached, traps are disabled, causing a negligible intrusion.

The use of a standard interface means that the monitor nodes can be easily added to the WSN during its development, improving system debugging and development time, or during the deployment stage to verify the correct operation of the sensor network. After removal, the monitor nodes can be reused in the monitoring of another WSN, and its cost may be recouped during repeated use in different monitoring studies.

In order to increase reusability, many interfaces may be used to communicate between the sensor node and monitor node. The performance of these interfaces may influence the overall intrusion caused to sensor node operation and, thus, affect the representativeness of the obtained results. In this paper several interfaces have been evaluated and characterized, with parallel or SPI interfaces being shown to be those which offer the best performance. However, as eighteen pins are necessary for parallel communication, which are not always available for monitoring purposes in sensor network nodes, it can be concluded that SPI, when available, is the better option in most cases.

The monitor was also applied to the monitoring of a real WSN application. Real measurements were obtained from a true operation in laboratory conditions. These measurements had associated timestamps and were stored in a SD non-volatile memory for later analysis.

As a result, it has been shown that low time interference permits a high trap generation rate. On the other hand, low code intrusion may be assumed in most common modern microcontrollers, even those with very low memory resources. Finally, the hybrid philosophy used allows the power consumption required to fulfill the monitoring tasks to be divided between the monitor node and sensor node, thus reducing the energy intrusion in the latter. This makes these techniques highly suitable for WSN monitoring (for designing, implementing, deploying or debugging purposes), but they may also be applied to the monitoring of many embedded systems.

Future research in monitor node development could include the use of communication interfaces, following the architecture in Figure 1, to provide greater support to the Interchange Layer. Many monitor nodes, and even passive monitors such as wireless network sniffers, could combine their data in a database. To achieve this goal it would be necessary to standardize the data format. Diverse monitoring data sources, even from different manufacturers, could then be joined to observe the behavior of the entire network. Real-time analysis and visualization of obtained data could also be improved, focusing on the use of standard tools.

A classification of the WSNs according to their acceptable monitor intrusion should be defined, and this intrusion index must condition the overall intrusion—number of captured events, communication interface used, *etc.*—which can be observed in a single monitoring campaign.

## References

[1] Patel M., Aggarwal A. Security Attacks in Wireless Sensor Networks: A Survey. Proceedings of the IEEE International Conference on Intelligent Systems and Signal Processing (ISSP).

[2] Mahapatro A., Khilar P.M. (2013). Fault Diagnosis in Wireless Sensor Networks: A Survey. IEEE Commun. Surv. Tutor..

[3] Rodrigues A., Camilo T., Silva J.S., Boavida F. (2012). Diagnostic Tools for Wireless Sensor Networks: A Comparative Survey. J. Netw. Syst. Manag..

[4] Rachedi A., Emary I., Ramakrishan S. (2013). Monitoring Mechanism for Wireless Sensor Networks: Challenges and Solutions. Wireless Sensor Networks.

[5] Schoofs A., O’Hare G.M.P., Ruzzelli A.G. (2012). Debugging Low-Power and Lossy Wireless Networks: A Survey. IEEE Commun. Surv. Tutor..

[6] Piqueras I., Campelo J.C., Ors R., Serrano J.J. Hybrid monitoring of wireless sensor networks. Proceedings of the IEEE International Conference on Wireless Information Technology and Systems (ICWITS).

[7] Navia M., Campelo J.C., Bonastre A., Serrano J.J. Low Intrusion Active Hybrid Monitor for Nodes of Sensor Networks. Proceedings of the Workshop on Innovation on Information and Communication Technologies (ITACA-WIICT).

[8] Tolle G., Culler D. Design of an Application-Cooperative Management System for Wireless Sensor Networks. Proceedings of the 2nd European Workshop on Wireless Sensor Networks (EWSN).

[9] Ramanathan N., Chang K., Kapur R., Girod L., Kohler E., Estrin D. Sympathy for the Sensor Network Debugger. Proceedings of the 3rd ACM Conference on Embedded Networked Sensor Systems.

[10] FAQ—TinyOS Wiki. http://tinyos.stanford.edu/tinyos-wiki/index.php/FAQ.

[11] Ringwald M., Römer K., Vialetti A. (2006). SNIF: Sensor Network Inspection Framework.

[12] Awad A., Nebel R., German R., Dressler F. On the Need for Passive Monitoring in Sensor Networks. Proceedings of the 11th EUROMICRO Conference on Digital System Design Architectures, Methods and Tools (DSD).

[13] Chen B., Peterson G., Mainland G., Welsh M. LiveNet: Using Passive Monitoring to Reconstruct Sensor Network Dynamics. Proceedings of the IEEE Conference on Distributed Computing in Sensor Systems (DCOSS).

[14] Kuang X., Shen J. SNDS: A Distributed Monitoring and Protocol Analysis System for Wireless Sensor Network. Proceedings of the 2010 Second International Conference on Networks Security, Wireless Communication and Trusted Computing (NSWCTC).

[15] Zhao Z., Wei H., Sun L. NSSN: A Network Monitoring and Packet Sniffing Tool for Wireless Sensor Networks. Proceedings of the 8th International Wireless Communications and Mobile Computing Conference.

[16] Garcia F., Andrade R., Oliveira C., de Souza J.N. (2014). EPMOSt: And Energy-Efficient Passive Monitoring System for Wireless Sensor Networks. Sensors.

[17] Liu Y., Liu K., Li M. (2010). Passive Diagnostics for Wireless Sensor Networks. IEEE/ACM Trans. Netw..

[18] Rost S., Balakrishnan H. Memento: A Health Monitoring System for Wireless Sensor Networks. Proceedings of the 3rd Annual IEEE Communications Society on Sensor and *Ad Hoc* Communications and Networks.

[19] Sundaram V., Eugster P., Zhang X. Lightweight tracing for wireless sensor networks debugging. Proceedings of the 4th International Workshop on Middleware Tools, Services and Run-Time Support for Sensor Networks (MidSens 09).

[20] Sommer P., Kusy B. Minerva: Distributed Tracing and Debugging in Wireless Sensor Networks. Proceedings of the 11th ACM Conference on Embedded Networked Sensor Systems (SenSys).

[21] Hossain M.S., Lee W.S., Raghunathan V. Spi-Snooper: A Hardware-Software Approach for Transparent Network Monitoring in Wireless Sensor Networks. Proceedings of the 8th IEEE/ACM/IFIP International Conference on Hardware/Software Codesign and Systems Synthesis.

[22] ISO/IEC Information Technology—Open Systems Interconnection—Basic Reference Model. http://www.iso.org/iso/home/store/catalogue_tc/catalogue_detail.htm?-csnumber=20269.

[23] STM32F051R8 ARM Cortex-M0 MCU. http://www.st.com/web/catalog/mmc/.

[24] CMSIS-Cortex Microcontroller Software Interface Standard. http://www.arm.com/products/processors/cortex-m/cortex-microcontroller-software-interface-standard.php.

[25] Keil MDK-ARM Version 5. http://www2.keil.com/mdk5/.

[26] 34405A Digital Multimeter, 5½ digit | Keysight (Agilent). http://www.keysight.com/en/pd-686884-pn-34405A/.

[27] Gharghan S., Nordin R., Ismail M. (2014). Energy-Efficient ZigBee-Based Wireless Sensor Network for Track Bicycle Performance Monitoring. Sensors.

[28] Molina-Garcia A., Fuentes J.A., Gómez-Lázaro E., Bonastre A., Campelo J.C., Serrano J.J. (2012). Development and Assessment of a Wireless Sensor and Actuator Network for Heating and Cooling Loads. IEEE Trans. Smart Grid.

[29] Lee D.S., Liu Y.H., Ling C.R. (2012). A Wireless Sensor Enabled by Wireless Power. Sensors.

[30] Cao Q., Abdelzaher T., Stankovic J., Whitehouse K., Luo L. Declarative tracepoints: A programmable and application independent debugging system for wireless sensor networks. Proceedings of the 6th ACM Conference on Embedded Network Sensor Systems.

